# Study on the related factors of TCM constitution and hemodynamics in patients with coronary heart disease

**DOI:** 10.3389/fcvm.2024.1383082

**Published:** 2024-03-11

**Authors:** Boyan Mao, Zhou Zhao, Minghui Wei, Xinzhu Liu, Ruoqi Zhao, Weipeng Zhang, Mengyao Duan

**Affiliations:** ^1^ School of Life Sciences, Beijing University of Chinese Medicine, Beijing, China; ^2^ Cardiac Surgery Department, Peking University People’s Hospital, Beijing, China; ^3^ School of Chinese Materia Medica, Beijing University of Chinese Medicine, Beijing, China; ^4^ Chengdu Techman Software Co. Ltd., Chengdu, China; ^5^ School of Traditional Chinese Medicine, Beijing University of Chinese Medicine, Beijing, China

**Keywords:** coronary artery, coronary heart disease, traditional Chinese medicine constitution, hemodynamics, modeling and simulation

## Abstract

**Background:**

The concepts of “individualization” and “preventive treatment” should be incorporated into the precise diagnosis and treatment of coronary heart disease (CHD). Both hemodynamics and Chinese medicine constitution studies align with these two concepts.

**Methods:**

This study utilized data from 81 patients with CHD, including 12 patients with balanced constitution (BC), 20 patients with blood stasis constitution (BSC), 17 patients with phlegm-dampness constitution (PDC), 15 patients with qi-deficiency constitution (QDC), and 17 patients with other constitutions. Clinical data provided information on the patients' blood property, heart function, degree of coronary stenosis, coronary hemodynamics, and so on. These parameters were compared between patients with balanced constitution vs. biased constitutions as well as between those with blood stasis constitution, phlegm-dampness constitution, and qi-deficiency constitution.

**Results:**

Compared to biased constitution (BC), patients with balanced constitution exhibited lower total cholesterol (TC) levels and low-density lipoprotein (LDL) levels. Additionally, they had lighter stenosis degrees in the Left anterior descending branch (LAD) and Left circumflex branch (LCX) branches. The hemodynamic condition of the LAD and LCX was better for those with balanced constitution; however there was no difference in heart function. Among the groups categorized by blood stasis, phlegm dampness or qi deficiency constituions, patients classified under phlegm dampness had higher levels of LDL compared to those classified under blood stasis or qi deficiency, while patients classified under qi deficiency had higher levels of blood glucose compared to those classified under blood stasis or phlegm dampness. Hemodynamic environments also differed among the LAD and LCX for each group but there were no significant differences observed in heart function or degree of coronary stenosis among these three groups.

**Conclusion:**

The balanced constitution demonstrates superior blood property, degree of coronary artery stenosis, and coronary hemodynamics compared to the biased constitution. Furthermore, among the three constitutions with CHD, variations in blood property and certain hemodynamic parameters are observed. These findings emphasize the significant clinical value of incorporating physical factors into the diagnosis and treatment of patients with CHD.

## 1 Introduction

Coronary heart disease is a prevalent cardiovascular disorder (1, 2) caused by stenosis in the coronary artery that impedes blood perfusion. The primary cause of this stenosis is the development of atherosclerotic plaque (3). Atherosclerotic plaques form within the coronary artery and subsequently obstruct it, leading to inadequate blood supply to the distal end of the artery and resulting in symptoms such as angina pectoris. In order to achieve precise diagnosis and treatment for coronary heart disease, it is essential to adopt the concepts of “individualization” and “preventive treatment”. The term “individualization” implies that even if patients exhibit similar examination results, their diagnosis and treatment decisions should still be tailored specifically for each individual. Similarly, “preventive treatment” emphasizes not only focusing on current coronary artery stenosis but also assessing future coronary blood flow transport capacity and preventing risk factors among potential patients with coronary heart disease. These two concepts are effectively implemented in hemodynamics theory and TCM constitution.

Hemodynamics is a scientific discipline that investigates the impact of blood flow and vascular physiology. Hemodynamic factors play a pivotal role in the onset and progression of coronary heart disease. Research has demonstrated that stable high wall shear stress (WSS) promotes the expression of endothelial cells' anti-atherogenic genes, whereas low WSS and high Oscillating shear index (OSI) facilitate the expression of atherogenic genes (4, 5). Analyzing the cardiovascular hemodynamic environment in patients enables an assessment of their risk for developing coronary heart disease, facilitating personalized prevention and treatment strategies for patients.

The constitution is a comprehensive and relatively stable trait of morphological structure, physiological function, and mental state that develops based on innate endowment and acquired factors. In traditional Chinese Medicine, the study of constitution characteristics, evolution rules, influencing factors, and classification standards aims to understand different constitutions starting from human beings. TCM has a complete theoretical and practical system for preventing, diagnosing, treating, rehabilitating, and preserving health in diseases. The balanced constitution is the most prevalent among the general population in China, comprising 32.14% according to epidemiological survey data conducted by academician Qi Wang across 9 provinces and cities with a sample size of 21,948 individuals. Meanwhile, the biased constitution accounts for 67.86%. Coronary heart disease (CHD) is a chronic disease with high incidence and risk where the concept of “arguing body to judge treatment” based on TCM constitution can significantly contribute to its diagnosis and treatment. Among the prevalent constitutional types observed in patients with CHD, the predominant three are blood stasis constitution, phlegm-dampness constitution, and qi-deficiency constitution, accounting for 20.96%, 18.46%, and 15.86% respectively (6). The TCM constitution is closely associated with the severity of coronary artery stenosis and the number of affected branches, as well as blood properties such as density, hemodynamic viscosity, and other cardiovascular characteristics.

Both hemodynamics and TCM constitution incorporate the principles of “personalized” and “preventive treatment” in the diagnosis and management of coronary heart disease. Simultaneously, they also examine CHD from a holistic perspective, considering patients' overall health status as well as local blood flow characteristics within the coronary artery. Consequently, this study aims to investigate the correlation between patients' constitution and hemodynamic factors associated with CHD, encompassing the relationship between constitution and blood properties, constitution and cardiac function, constitution and morphological structure of coronary arteries, as well as constitution and coronary hemodynamics.

## 2 Method

### 2.1 Patient data collection

The clinical patient data utilized in this study were obtained from the Heart Center of Peking University People's Hospital between 2021 and 2023. The following are the selection criteria for inclusion by our patients.

#### 2.1.1 Inclusion and exclusion criteria of patients

Inclusion criteria:

① Age ≥18 and ≤90:
② Completed coronary CTA, echocardiography and blood routine examination in the hospital;
③ The informed consent shall be signed by the applicant or his/her immediate family member.
④ The TCM Constitution Classification and Judgment Scale should be filled in truthfully under the guidance of TCM doctors.

Exclusion criteria:

① Age <18 years or >90 years;
② Received coronary stent or coronary artery bypass graft surgery;
③ Unclear consciousness, can not express subjective symptoms and psychiatric patients;
④ Accompanied by more than one serious secondary progressive malignant tumor or other serious wasting disease.

Coronary CTA enables the acquisition of the number, stenosis rate, and stenosis length for each branch of the LAD, LCX, and right coronary artery (RCA). Additionally, it facilitates 3D reconstruction of the coronary artery to calculate hemodynamics for each vessel. Echocardiography was employed to measure ejection fraction (EF), left ventricular end-diastolic diameter (LVEDD), and left atrial diameter (LA). Blood tests were conducted to determine TC levels, LDL levels, and blood glucose levels.

TCM constitution identification was accomplished by guiding patients through completion of the *TCM Constitution Classification and Judgment Scale*. Patients answered all questions on a 5-point scale within this questionnaire. Original scores were calculated alongside transformation scores to evaluate each patient's constitution type. In cases where patients exhibited more than two biased constitution, their main constitution type was identified based on higher transformation scores. A healthy constitution was defined as balanced while other constitutions were classified as biased constitution. Among patients with CHD, common constitution types included blood stasis constitution, phlegm-dampness constitution, and qi-deficiency constitution. This paper compares different constitutions in two aspects: firstly comparing balanced vs. biased constitution; secondly comparing blood stasis constitution against phlegm-dampness constitution and qi-deficiency constitution.

### 2.2 Construction of coronary hemodynamic model and data collection

#### 2.2.1 3D coronary artery model construction

In this study, a 0D-3D coupled multi-scaled modeling method was utilized to construct a coronary hemodynamic model. The coronary artery CTA data were used for 3D reconstruction, and tetrahedral grids were employed for meshing. All grids passed sensitivity analysis. Then, the vessel properties and blood properties were set as rigid wall, the blood was set as incompressible viscous Newtonian fluid, the density of the blood was set as 1,050 kg/m^3^, and the kinetic viscosity was set as 0.0035 Pa·s. The governing equation of blood flow is Navier-Stokes equation (N-S equation), and the flow is set to laminar flow.

#### 2.2.2 Construction of coronary 0D-3D coupled multi-scaled model

**Figure 1:**
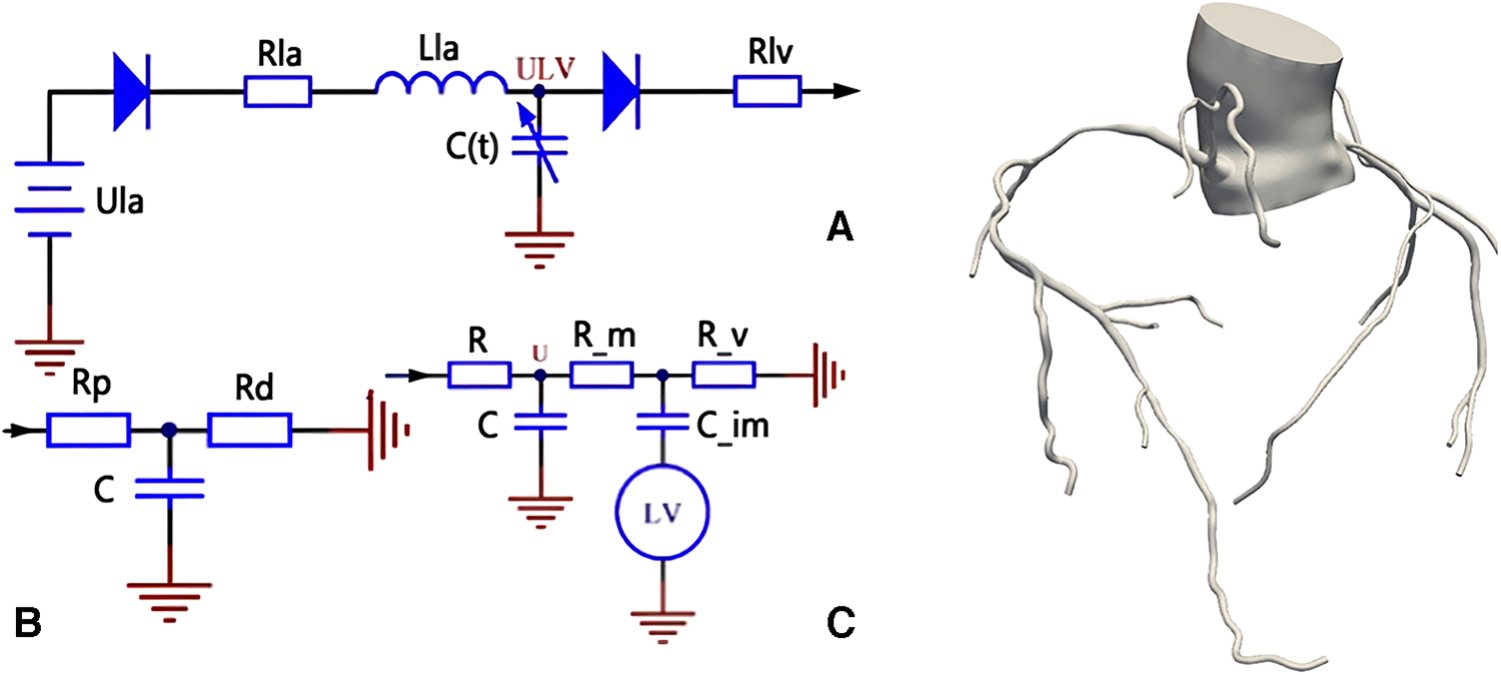
Coronary 0D-3D coupled multi-scaled model. (**A**) is the heart module, which provides boundary conditions for the aortic inlet of the model; (**B**) is the systemic circulation module, which provides boundary conditions for the aortic outlet of the model; and (**C**) is the coronary module, which provides boundary conditions for the coronary outlet of the model.

In this study, the lumped parameter model (also known as 0D model) was used to provide boundary conditions for the 3D model, so it is necessary to design 0D model of different structures according to the parts connected by the inlet and outlet of the 3D model. The inlet of the 3D model was the heart, and its outlet included the distal of the aorta and its bifurcation vessels, as well as the distal of the coronary vessels. Therefore, the 0D model for this study consists of three modules: the heart module, the systemic circulation module and the coronary circulation module. The design structure of these three modules is shown in Figure 1.

For the heart module, its structure is shown in Part A of the figure. In order to provide a boundary condition for the aortic inlet of the 3D model, our heart model only contains the left heart part, and omits the right heart part. In the left heart, the blood enters the left ventricle through the left atrium and the mitral valve. The left ventricle pushes the blood out of the aorta and then the systemic circulation is completed. Ula in part A of the picture is a constant voltage source, representing the pressure in the left atrium, This is because the stress in the left atrium is small and does not fluctuate significantly with the cardiac cycle, so a constant voltage source can be used instead. The two diodes represent the mitral valve and the aortic valve from left to right respectively, which ensures the unidirectional conduction of the current; the resistance Rla and the inductance Lla represent the blood flow resistance and the blood flow inertia flowing through the mitral valve respectively; the resistance Rlv represents the blood flow resistance flowing through the aortic valve. *C*(*t*) is a time-varying capacitance that reflects the periodic contraction and relaxation of the left ventricle over time, and its value is regulated by the pressure-volume relationship of the left ventricle.

For the systemic circulation module, it is shown in part B of the figure. The resistance R_p_ represents the resistance of arterial blood flow, the resistance R_d_ represents the sum of the resistance of the arterial end, the microcirculatory system and the venous system, and the capacitance C represents the elasticity of the arterial blood vessel.

For the coronary module, unlike other vessels, the coronary vessel reaches its peak blood flow during diastole. In order to simulate the special phenomenon of coronary artery, the lumped parameter model of coronary artery needs to consider the influence of myocardial contraction, and the general method is to add a pressure source synchronous with the ventricular pressure in the lumped parameter model. Part C in the figure is the structural diagram of the coronary lumped parameter model. The resistance R represents the coronary blood flow resistance, the resistance R_m represents the coronary microcirculation blood flow resistance, and the resistance R_v represents the venous blood flow resistance. Capacitor C represents the coronary artery. The capacitance C_im represents the vascular compliance of coronary microcirculation. The lower end of the capacitance C_im is connected with a voltage source which represents the extrusion of the myocardial motion to the coronary artery, and the change of the value follows the change of the pressure of the left ventricle.

After determining the structure of the lumped parameter model, the next task is to select the appropriate parameters for each component of the model. In this paper, the genetic algorithm is used to optimize the parameters, and the problem of matching the parameters of the components with the physiological data is solved with the patient's personalized physiological characteristics data as the target (9).

Using the data of systolic pressure, diastolic pressure, heart rate and cardiac output of normal people, the aortic pressure waveform and cardiac output waveform of normal people are fitted as two optimized target waveforms. Based on the research of Kim (8), the parameter values of the lumped parameter model are adjusted manually to the extent that the output waveform matches the target waveform, and the parameters at this time are used as the reference values of the subsequent personalized parameters. In this process, two important points should be noted: (1) the total coronary flow accounts for 4% of the cardiac output, and the left coronary flow and the right coronary flow account for 60% and 40% of the total coronary flow, respectively; (2) the blood flow of the coronary branches accounts for 2.7 power.

Then, the 3D model and 0D model were coupled with specific interface conditions and coupling algorithms, and the coronary 0D-3D coupled multi-scaled model was constructed as shown in Figure 1. The 0D model provides the flow conditions at the entrance and the pressure boundary conditions at the exit for the 3D model. After calculation, the 3D model is the return pressure at the entrance and the discharge at the exit of the 0D model. The specific construction process of 0D-3D coupled multi-scaled model can be referred to the previous research of our research group (9, 10–12).

#### 2.2.3 Extraction of hemodynamic results

After constructing the multi-scaled model and completing the calculation, the hemodynamic results of the three main branches of the coronary artery were extracted, including WSS, wall shear stress gradient (WSSG), OSI and other parameters. At the same time, the 0D model was adjusted to make the afterload become 0.24 times of the resting state to simulate the maximum hyperemia state. The model was calculated under this state, and the FFR (fractional flow reserve) at the distal end of the three main coronary branches was extracted (13, 14).

### 2.3 Construction of coronary artery repair model

**Figure 2:**
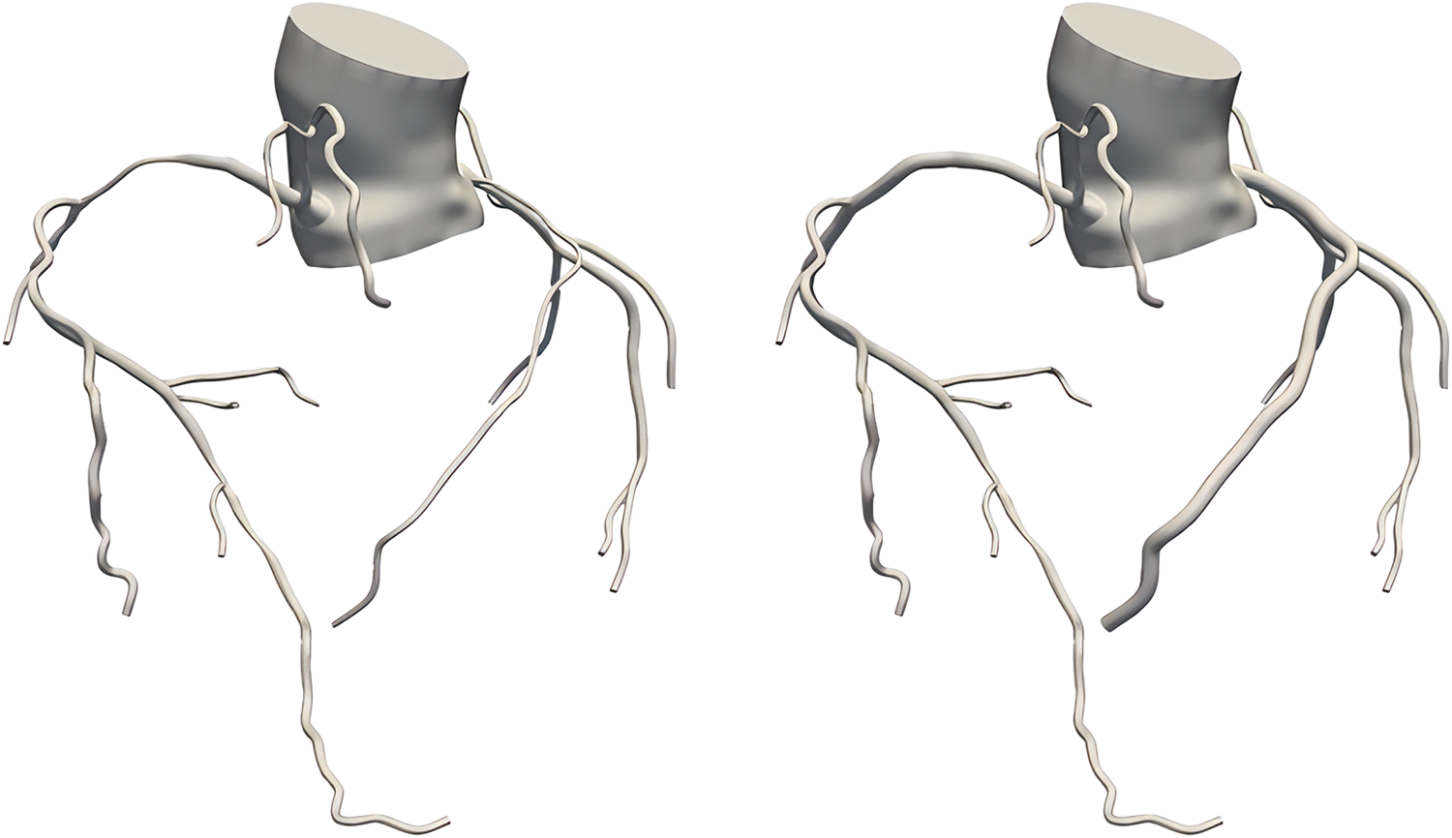
True coronary artery model (left), coronary artery repair model (right).

Traditional Chinese medicine constitution is good at analyzing the vulnerability and tendency of different people to diseases, which emphasizes “prevention before disease onset”. The effect of hemodynamics on blood vessels can also be revealed through a certain period of time. In order to study the long-term development of coronary heart disease in patients with different constitutions, a coronary artery repair model was constructed based on a real coronary artery model, as shown in Figure 2.

In this study, the diameter of the anterior and posterior ends of the coronary artery stenosis was averaged as the reference diameter, and then the coronary artery stenosis was repaired using virtual surgical software to make its diameter into the reference diameter, so that the coronary artery repair model without stenosis was obtained. The coronary artery repair model was used to simulate the state of patients when they were “normal”, and to study whether there were differences in hemodynamics between patients with different constitutions when they were normal. The subsequent modeling methods and the extraction process of hemodynamic results of the coronary repair model were consistent with the real coronary model.

### 2.4 Statistical method

One-way analysis of variance (ANOVA) was utilized to compare the differences between two groups, namely balanced constitution and biased constitution, as well as three groups including blood stasis constitution, phlegm-dampness constitution, and qi-deficiency constitution. The statistical analysis was conducted using SPSS software. This method effectively enables us to explore the variations in mean values among different groups and subsequently verify our research hypothesis. Prior to conducting the analysis, normal distribution and homogeneity of variance were confirmed for all data. Results were expressed as means with standard deviations. *P*-value less than 0.05 indicated a significant difference between groups.

## 3 Results

### 3.1 Patient baseline data and constitution type

The data utilized in this study were obtained from the Heart Center of Peking University People's Hospital during the period from 2021 to 2023, encompassing a total of 81 patients. The baseline data and constitution classifications are presented in Table 1.

**Table 1:** Baseline data and constitution types of patients.

Number of patients	81
Height (cm)	167.3 ± 7.5
Age	62.9 ± 9.3
Weight (kg)	72.5 ± 14.1
Gender	Male 61/female 20
balanced quality	12
Blood stasis	20
Phlegm dampness	17
Qi-deficiency quality	15
Other constitution	17

### 3.2 Comparison of blood properties and cardiac function in patients with different TCM constitutions

In this study, TC, LDL, blood glucose, left ventricular ejection fraction (LVEF), LVEDD, LA and other parameters were obtained according to blood examination and cardiac ultrasound examination of patients, and their comparison in different TCM constitution groups was as follows. For the grouping of balanced constitution and biased constitution, the results are shown in Table 2.

**Table 2:** Comparison of blood properties and cardiac function between balanced and biased constitution.

	Balanced constitution	Biased constitution	*P*-value
TC (mmol/L)	3.258 ± 0.364	3.828 ± 0.888	<0.05
LDL (mmol/L)	1.903 ± 0.344	2.393 ± 0.660	<0.05
Blood glucose (mmol/L)	5.378 ± 0.930	6.483 ± 2.099	0.078
LVEF (%)	62.225 ± 13.231	63.812 ± 8.896	0.599
LVEDD (mm)	50.383 ± 7.938	47.712 ± 11.680	0.449
LA (mm)	39.442 ± 5.332	36.359 ± 8.450	0.227

Table 2 demonstrates significant disparities in TC and LDL levels between patients with balanced and biased constitution. The balanced constitution group exhibited lower levels of both TC and LDL, whereas the biased constitution group displayed higher levels of both biomarkers. These differences were statistically significant. Furthermore, there was a discernible distinction in blood glucose levels between patients with balanced and biased constitution. However, this disparity did not meet the criteria for statistical significance. Notably, no significant variations were observed between the two groups regarding other cardiac function measures such as LVEF, LVEDD, and LA. For the groups of blood stasis, phlegm dampness, and qi deficiency, the results are shown in Table 3.

**Table 3:** Comparison of blood property and cardiac function of blood stasis constitution, phlegm dampness constitution and qi-stagnation constitution qualities.

	blood stasis constitution	Phlegm dampness	Qi-deficiency	*P*-value
TC (mmol/L)	3.974 ± 0.766	4.191 ± 1.228	3.635 ± 0.574	0.228
LDL (mmol/L)	2.383 ± 0.619	2.825 ± 0.831	2.105 ± 0.483	<0.05
Blood glucose (mmol/L)	5.901 ± 1.412	6.355 ± 1.622	8.139 ± 3.263	<0.05
LVEF (%)	63.150 ± 7.797	65.159 ± 7.745	64.196 ± 11.302	0.792
LVEDD (mm)	45.380 ± 14.337	49.776 ± 4.201	44.000 ± 16.236	0.399
LA (mm)	35.375 ± 11.755	37.459 ± 5.302	33.940 ± 8.786	0.555

According to Table 3, significant differences were observed in LDL and blood glucose levels among patients with blood stasis constitution, phlegm-dampness, and qi-deficiency constitutions. Post hoc multiple comparisons revealed that the LDL level was significantly higher in patients with phlegm-dampness constitution compared to those with blood stasis constitution and qi-deficiency constitutions. Additionally, the blood glucose level was significantly higher in patients with qi-deficiency constitution compared to those with blood stasis constitution and phlegm-dampness constitutions. However, no significant differences were found in TC levels and various cardiac function indexes among the three constitutional types.

### 3.3 To compare the severity of coronary artery stenosis among patients with different traditional Chinese medicine constitutions

In this study, all patients were examined for the three main branches of the coronary artery. The degree of coronary artery stenosis was determined based on CTA data, which primarily included the maximum stenosis rate of each coronary artery, the corresponding length of stenosis at the maximum rate, and the FFR measurement at the distal end of each coronary artery. Table 4 presents the findings for both groups with a balanced constitution and biased constitution.

**Table 4:** Comparison of the degree of coronary artery stenosis between the balanced constitution and the biased constitution.

	Balanced constitution	Biased constitution	*P*-value
Maximum stenosis rate of LAD	0.579 ± 0.339	0.769 ± 0.192	<0.05
Length of LAD stenosis (cm)	2.960 ± 2.465	3.932 ± 2.369	0.196
LAD FFR	0.788 ± 0.184	0.487 ± 0.172	<0.05
LCX maximum stenosis rate	0.450 ± 0.342	0.609 ± 0.316	0.117
LCX stenosis length (cm)	2.538 ± 2.866	2.121 ± 1.937	0.525
LCX FFR	0.761 ± 0.167	0.628 ± 0.204	<0.05
Maximum RCA stenosis	0.621 ± 0.367	0.712 ± 0.278	0.319
RCA stenosis length (cm)	2.306 ± 2.711	4.104 ± 3.931	0.133
RCA FFR	0.688 ± 0.220	0.592 ± 0.214	0.157

According to Table 4, there were statistically significant differences observed between patients with balanced and biased constitution in terms of the maximum rate of stenosis in LAD, as well as the FFR values for both LAD and LCX. Specifically, the maximum rate of stenosis in LAD was significantly higher in the biased constitution group compared to the balanced constitution group. Additionally, the FFR value for LAD was significantly lower in the biased constitution group than in the balanced constitution group. These findings suggest a higher likelihood of coronary stenosis occurring specifically within LAD among patients with a biased constitution. Similarly, LCX FFR values were significantly lower in the biased group compared to those with a balanced constitution, indicating a more severe degree of coronary stenosis within LCX among patients with a biased constitution.

The results for the groups with blood stasis constitution, phlegm dampness, and qi-deficiency constitution are presented in Table 5. It is evident that there were no statistically significant differences observed in the degree of stenosis among the three coronary arteries across these groups.

**Table 5:** Comparison of the degree of coronary artery stenosis of blood stasis, phlegm dampness and qi deficiency.

	Blood stasis constitution	Phlegm dampness	Qi- deficiency	*P*-value
Maximum stenosis rate of LAD	0.755 ± 0.161	0.799 ± 0.206	0.75 ± 0.218	0.734
LAD stenosis length (cm)	4.087 ± 1.687	3.528 ± 1.759	4.42 ± 2.839	0.477
LAD FFR	0.437 ± 0.161	0.470 ± 0.173	0.52 ± 0.182	0.362
LCX maximum stenosis	0.595 ± 0.294	0.535 ± 0.333	0.68 ± 0.235	0.345
LCX stenosis length (cm)	2.102 ± 1.832	1.621 ± 1.357	2.54 ± 2.171	0.358
LCX FFR	0.562 ± 0.216	0.676 ± 0.204	0.64 ± 0.143	0.197
Maximum RCA stenosis	0.740 ± 0.233	0.668 ± 0.311	0.63 ± 0.318	0.544
RCA stenosis length (cm)	4.322 ± 3.977	2.528 ± 1.928	5.27 ± 5.728	0.162
RCA FFR	0.543 ± 0.221	0.656 ± 0.187	0.60 ± 0.240	0.290

### 3.4 Comparison of coronary hemodynamics in patients with different TCM constitutions

The coronary 0D-3D coupled multi-scaled model was constructed using coronary CTA in this study, and the hemodynamic parameters WSS, WSSG, and OSI were extracted from LAD, LCX, and RCA after calculation. Based on the coronary artery repair model, the hemodynamic parameters in the “normal” state were calculated and extracted. Table 6 presents the results for grouping based on balanced constitution and biased constitution.

**Table 6:** Comparison of coronary hemodynamics between balanced constitution and biased constitution.

		Balanced constitution	Biased constitution	*P*-value
True model of coronary artery	LAD-WSS (dyn/cm^2^)	51.771 ± 27.801	70.627 ± 31.622	<0.05
	LAD-WSSG (dyn/cm^3^)	208.616 ± 133.624	317.467 ± 157.940	<0.05
	LAD-OSI	0.0098 ± 0.0071	0.0101 ± 0.0154	0.945
	LCX-WSS (dyn/cm^2^)	54.326 ± 34.009	80.509 ± 46.167	<0.05
	LCX-WSSG (dyn/cm^3^)	246.186 ± 203.123	389.418 ± 247.637	<0.05
	LCX-OSI	0.0059 ± 0.0037	0.0073 ± 0.0081	0.574
	RCA-WSS (dyn/cm^2^)	51.515 ± 29.091	67.563 ± 32.761	0.116
	RCA-WSSG (dyn/cm^3^)	241.061 ± 163.609	316.871 ± 178.248	0.173
	RCA-OSI	0.0070 ± 0.0079	0.0075 ± 0.0085	0.831
Model of coronary artery repair	LAD-WSS (dyn/cm^2^)	65.016 ± 26.717	64.634 ± 30.798	0.968
	LAD-WSSG (dyn/cm^3^)	249.951 ± 102.668	246.670 ± 123.256	0.931
	LAD-OSI	0.0086 ± 0.0059	0.0091 ± 0.0090	0.839
	LCX-WSS (dyn/cm^2^)	71.081 ± 47.490	71.427 ± 38.576	0.978
	LCX-WSSG (dyn/cm^3^)	299.826 ± 194.996	316.860 ± 190.293	0.776
	LCX-OSI	0.0053 ± 0.0052	0.0071 ± 0.0076	0.412
	RCA-WSS (dyn/cm^2^)	63.333 ± 30.792	64.396 ± 34.051	0.920
	RCA-WSSG (dyn/cm^3^)	245.235 ± 118.169	257.592 ± 147.201	0.784
	RCA-OSI	0.0057 ± 0.0055	0.0061 ± 0.0089	0.893

In the real coronary model, significant differences in WSS and WSSG were observed between patients with a balanced constitution and those with a biased constitution, as presented in Table 6. The WSS and WSSG values were higher in the biased constitution group compared to the balanced constitution group, particularly in the LAD and LCX coronary branches. No significant differences were found for OSI among different coronary artery branches. However, no significant variations in hemodynamic parameters of each coronary artery were observed between the two groups in the coronary repair model.

Table 7 presents the results for the groups categorized by blood stasis, phlegm dampness, and qi-deficiency. In the realistic coronary model, significant differences were observed in WSS among the LAD branches across the three groups. Post hoc multiple comparisons revealed that the LAD-WSS of the blood stasis type was lower than that of both the phlegm dampness type and qi-deficiency type. Significant differences were also found in OSI among LCX branches within these three groups. According to *post hoc* multiple comparisons, the LCX-OSI of the qi deficiency type was lower compared to both blood stasis and phlegm dampness types. No significant differences were observed among these three groups for other hemodynamic parameters. However, in terms of coronary repair models, all hemodynamic parameters showed no significant variations between groups.

**Table 7:** Comparison of coronary hemodynamics of blood stasis, phlegm dampness and qi deficiency.

		Blood stasis constitution	Phlegm dampness	Qi-deficiency	*P*-value
True model of coronary artery	LAD-WSS (dyn/cm^2^)	54.765 ± 23.501	78.511 ± 30.647	81.439 ± 39.171	<0.05
	LAD-WSSG (dyn/cm^3^)	259.22 ± 122.661	336.62 ± 164.750	365.2 ± 190.434	0.127
	LAD-OSI	0.0149 ± 0.0266	0.0086 ± 0.0055	0.0083 ± 0.0095	0.451
	LCX-WSS (dyn/cm^2^)	78.922 ± 45.708	78.266 ± 53.599	90.421 ± 50.787	0.742
	LCX-WSSG (dyn/cm^3^)	364.27 ± 210.795	378.05 ± 273.240	472.2 ± 317.341	0.457
	LCX-OSI	0.0103 ± 0.0085	0.0099 ± 0.0131	0.0040 ± 0.0029	< 0.05
	RCA-WSS (dyn/cm^2^)	58.025 ± 34.508	68.218 ± 33.687	77.667 ± 35.127	0.254
	RCA-WSSG (dyn/cm^3^)	262.35 ± 165.519	327.63 ± 176.788	361.3 ± 215.026	0.276
	RCA-OSI	0.0092 ± 0.0093	0.0073 ± 0.0067	0.0072 ± 0.0124	0.790
Model of coronary repair	LAD-WSS (dyn/cm^2^)	50.240 ± 32.746	61.719 ± 31.486	72.978 ± 26.799	0.105
	LAD-WSSG (dyn/cm^3^)	186.37 ± 129.582	230.45 ± 121.661	282.9 ± 104.689	0.073
	LAD-OSI	0.0112 ± 0.0134	0.0104 ± 0.0091	0.0073 ± 0.0041	0.504
	LCX-WSS (dyn/cm^2^)	70.103 ± 49.109	65.529 ± 44.340	72.29 ± 29.021	0.899
	LCX-WSSG (dyn/cm^3^)	306.344 ± 244.391	282.262 ± 203.807	331.107 ± 161.624	0.807
	LCX-OSI	0.0080 ± 0.0091	0.0102 ± 0.0100	0.0057 ± 0.0041	0.322
	RCA-WSS (dyn/cm^2^)	54.870 ± 38.976	62.137 ± 35.038	71.825 ± 33.256	0.396
	RCA-WSSG (dyn/cm^3^)	212.11 ± 159.639	258.18 ± 158.509	290.098 ± 152.239	0.344
	RCA-OSI	0.0101 ± 0.0152	0.0051 ± 0.0032	0.0041 ± 0.0029	0.150

## 4 Discussion

### 4.1 TCM constitution and coronary heart disease

The TCM constitution theory classifies patients into nine fundamental constitution types:balanced constitution, qi-deficiency constitution, yin-deficiency constitution, yang-deficiency constitution, phlegm-dampness constitution, blood stasis constitution, damp-heat constitution, qi-stagnation constitution, and inherited special constitution. Each constitutional type possesses distinct characteristics in terms of physical property, physiological traits, psychological features, pathological response states, and disease tendencies. The balanced constitution represents a state of optimal health. The remaining eight constitutions are referred to as biased constitution which impact the overall health status of patients as well as their susceptibility and predisposition to diseases. Among the general population in China, the most prevalent constitutional type is the balanced constitution accounting for 32.14%. Biased constitution account for 67.86%, with the top three being qi-deficiency Constitution, damp-heat constitution, and yang-deficiency constitution (6). Balanced and biased constitution serve as crucial classifications in constitutional studies representing healthy patients vs. those with compromised health conditions. Among the common constitution types of patients with CHD, the three most prevalent are blood-stasis constitution, phlegm-dampness constitution, and qi-deficiency constitution, accounting for 20.96%, 18.46%, and 15.86% respectively (6). Among these three CHD-prone constitutions, blood stasis showed the most severe degree of coronary artery stenosis and higher levels of blood lipids, making it more susceptible to hypertension. phlegm-dampness exhibited a relatively better heart function performance but also had a higher degree of coronary artery stenosis and elevated blood lipid levels, making it highly prone to hypertension. Qi-deficiency demonstrated a more serious degree of coronary artery stenosis along with higher blood glucose levels and poor vascular compliance (15–18). Patients with other biased constitutions accounted for a small proportion among those with CHD, and their diagnostic characteristics for this condition were relatively weak; therefore, they were not included in this study.

Many scholars have conducted targeted and highly detailed studies on the correlation between TCM constitution and CHD. These research findings have laid a preliminary foundation for our team's work, enabling us to further contemplate the relationship between constitution and CHD, as well as conduct more in-depth research.

In terms of the relationship between TCM constitution and coronary artery morphology, numerous scholars have discovered significant variations in coronary artery morphology among patients with different constitutions suffering from CHD, including differences in the degree of stenosis and number of affected branches (15). Overall, biased constitutions exhibit greater degrees of coronary artery stenosis compared to balanced constitutions. The qi-deficiency constitution, blood-stasis constitution, phlegm-dampness constitution, and other CHD-prone constitutions demonstrate higher levels than other biased constitutions. Positive syndrome constitutions (blood-stasis constitution, phlegm-dampness constitution) surpass deficiency syndrome constitutions (qi-deficiency constitution, yang-deficiency constitution), with blood-stasis constitutional types exceeding phlegm-dampness constitutional types.

Regarding TCM constitution and blood properties, certain constituents such as total cholesterol, triglyceride, low-density lipoprotein cholesterol (LDL-C) are considered risk factors for increased blood viscosity and vascular blockage. Scholars have observed that specific constitutions can elevate blood lipids and glucose levels while inducing viscous blood consistency leading to circulatory obstruction, impaired hemodynamics, and subsequent vascular blockages (16–18). Generally speaking, Yin deficiency and qi-deficiency constitute primarily contribute to elevated blood glucose levels.

However, most researchers have solely focused on the association between constitution, blood properties, and coronary artery stenosis without conducting a multi-faceted comparison based on a batch of data. No scholars have explored the relationship between constitution and FFR, WSS, WSSG, OSI—parameters that can characterize the current or future blood transport function of the coronary artery. Our team believes that delving into this area of research may yield greater value. Therefore, we aim to further investigate the relationship between constitution and hemodynamics in TCM building upon previous scholarly research.

Introducing TCM constitution into the study of coronary hemodynamics holds significant importance. The constitutional theory of TCM serves as an essential basis for diagnosing and treating CHD within this field. Patients with different constitutions exhibit distinct manifestations of CHD requiring varying treatment methods. Approaching treatment from a constitutional perspective aligns with the concept of “preventive treatment for diseases” by focusing on preventing illness before it occurs or progresses while also preventing relapse after recovery. The combination of TCM constitution analysis with hemodynamic studies has demonstrated great value in diagnosing and treating patients with CHD.

### 4.2 Traditional Chinese medicine constitution and blood properties, heart function, coronary artery morphology and structure

The study revealed no disparity in cardiac function between patients with a balanced constitution and those with a biased constitution. However, significant variations were observed in blood property and the extent of coronary artery stenosis, potentially linked to the metabolic and physiological characteristics associated with each constitution type. The balanced constitution group exhibited lower levels of TC and LDL, both crucial risk factors for cardiovascular disease, suggesting better cardiovascular health. Conversely, patients with a biased constitution displayed higher levels of these measures, indicating an elevated risk for cardiovascular disease. Furthermore, although not statistically significant, the discrepancy in glucose levels between patients with a balanced or biased constitution is noteworthy due to hyperglycemia's association as another risk factor for cardiovascular disease. The degree of coronary artery stenosis serves as an essential indicator to assess the severity of cardiovascular disease. Significant disparities were found in the maximum LAD stenosis rate, LAD FFR, and LCX FFR between patients with balanced constitution compared to those with biased constitution; this suggests that the biased constitution group is more prone to developing coronary stenosis. This outcome may be attributed to risk factors such as dyslipidemia and inflammatory response prevalent among patients with a biased constitution.

For the grouping of blood stasis constitution, phlegm dampness and qi-deficiency constitutions, this study found that there were significant differences among the three constitutions in the levels of LDL and blood glucose, but no significant differences in TC and various cardiac function indexes were found among the three constitutions. Specifically, the LDL level of the phlegm-dampness constitution was significantly higher than that of the blood stasis constitution and the qi-deficiency constitution, and the blood glucose level of the qi-deficiency constitution was significantly higher than that of the blood stasis constitution and the phlegm-dampness constitution, which suggested that the phlegm-dampness constitution population faced the risk of hyperlipidemia, and the qi-deficiency constitution population faced the risk of hyperglycemia. There was no significant difference in heart function and degree of coronary artery stenosis among the three constitutions. This indicates that the differences among the three constitutions of CHD are only in the properties of blood, but there is no significant difference in the structure and function of the heart and coronary vessels.

### 4.3 Constitution of traditional Chinese medicine and coronary hemodynamics

For the groups with mild and biased constitution, significant differences were observed in WSS and WSSG, but not in OSI in the real coronary model. Specifically, WSS and WSSG were higher in the biased group compared to the mild group, particularly in the LAD and LCX coronary branches. This finding is consistent with a higher degree of stenosis observed in patients with a biased constitution compared to those with a balanced constitution. Blood flow velocity increases at the site of stenosis, and as stenosis severity worsens, this increase becomes more pronounced, leading to an elevation in WSS within hemodynamics. The increase in WSS occurs locally at the site of stenosis followed by a rapid decrease in flow velocity reflected by WSSG, resulting in an overall increase of adverse hemodynamic factors that can contribute to further aggravation of atherosclerosis. In the coronary repair model, no differences were found between both groups regarding all hemodynamic parameters across all coronary branches. This indicates that there are no disparities concerning hemodynamics between patients with balanced constitution and those with biased constitution under “normal” conditions. Furthermore, it suggests that adverse hemodynamic environments are not solely generated due to inherent morphological structures associated with biased constitution themselves. From a purely hemodynamic perspective alone, patients with biased constitution do not exhibit a greater predisposition for developing coronary artery stenosis compared to those with balanced constitution. However, it should be noted that these findings do not take into account potential variations related to blood properties.

For the groups with blood stasis constitution, phlegm-dampness constitution, and qi-deficiency constitution in the realistic coronary model, this study observed that the WSS of the LAD branch in patients with blood stasis constitution was significantly lower compared to those with phlegm-dampness constitution and qi-deficiency constitution. Additionally, the WSSG was slightly smaller for patients with blood stasis constitution compared to the other two constitutions, approaching values similar to those of patients with a balanced constitution. These findings suggest that patients with blood stasis constitution exhibit a hemodynamic environment in their LAD branch comparable to that of patients with a balanced constitution. However, it should be noted that the OSI of the LCX branch in patients with qi-deficiency quality is lower than that of those with blood stasis and phlegm-dampness constitution. OSI is considered an unfavorable hemodynamic factor indicating better hemodynamic conditions for patients with qi-deficiency quality in their LCX branch. In contrast, no significant differences were observed among all groups regarding hemodynamic parameters across all coronary branches within the coronary artery repair model. This suggests that there are no variations in hemodynamics related to blood stasis, phlegm-dampness, and qi-deficiency under “normal” conditions.

### 4.4 Limitation

The present study has the following limitations: (1) The sample size selected for the study was insufficient, with data collected from only 81 eligible patients, resulting in small sample sizes for each constitution after grouping. Particularly for patients with a balanced constitution, the proportion of such patients among coronary heart disease patients is low and significantly differs from biased constitution data. (2) During hemodynamic modeling and simulation, only the personalized 3D model of the coronary artery was considered, while other conditions, especially blood properties based on different constitutions, were not taken into account. Current results indicate significant variations in blood properties between different constitutions–whether it be balanced constitution and partial constitution or blood stasis constitution, phlegm-dampness constitution, and qi-deficiency constitution. However, our model assumes uniform blood density and kinetic viscosity which may introduce certain deviations in hemodynamic outcomes.

## 5 Conclusion

Through this study, we investigated the association between balanced constitution and biased constitution, as well as the relationship among blood stasis constitution, phlegm-dampness constitution, and qi-deficiency constitution in terms of blood property, cardiac function, degree of coronary stenosis, and coronary hemodynamics. The findings of this study revealed that patients with a balanced constitution exhibited superior blood property compared to those with a biased constitution, along with reduced degree of coronary artery stenosis and improved coronary hemodynamics. Furthermore, variations in blood properties and certain hemodynamic parameters were observed among the three constitutions in patients with CHD. These results emphasize the significant clinical value of incorporating physical factors into the diagnosis and treatment of patients with coronary heart disease.

## Data Availability

The raw data supporting the conclusions of this article will be made available by the authors, without undue reservation.
